# Supplementary material for: Onychomycosis in Two Populations with Different Socioeconomic Resources in an Urban Nucleus: A Cross-Sectional Study
Source: J Fungi (Basel). 2022 Sep 24;8(10):1003. doi: 10.3390/jof8101003 (PMC9604941; doi:10.3390/jof8101003)
Supplement: Supplementary file 1 [file jof-08-01003-s001.zip › jof-1928231-supplementary.pdf]

- **USUARIO 1**

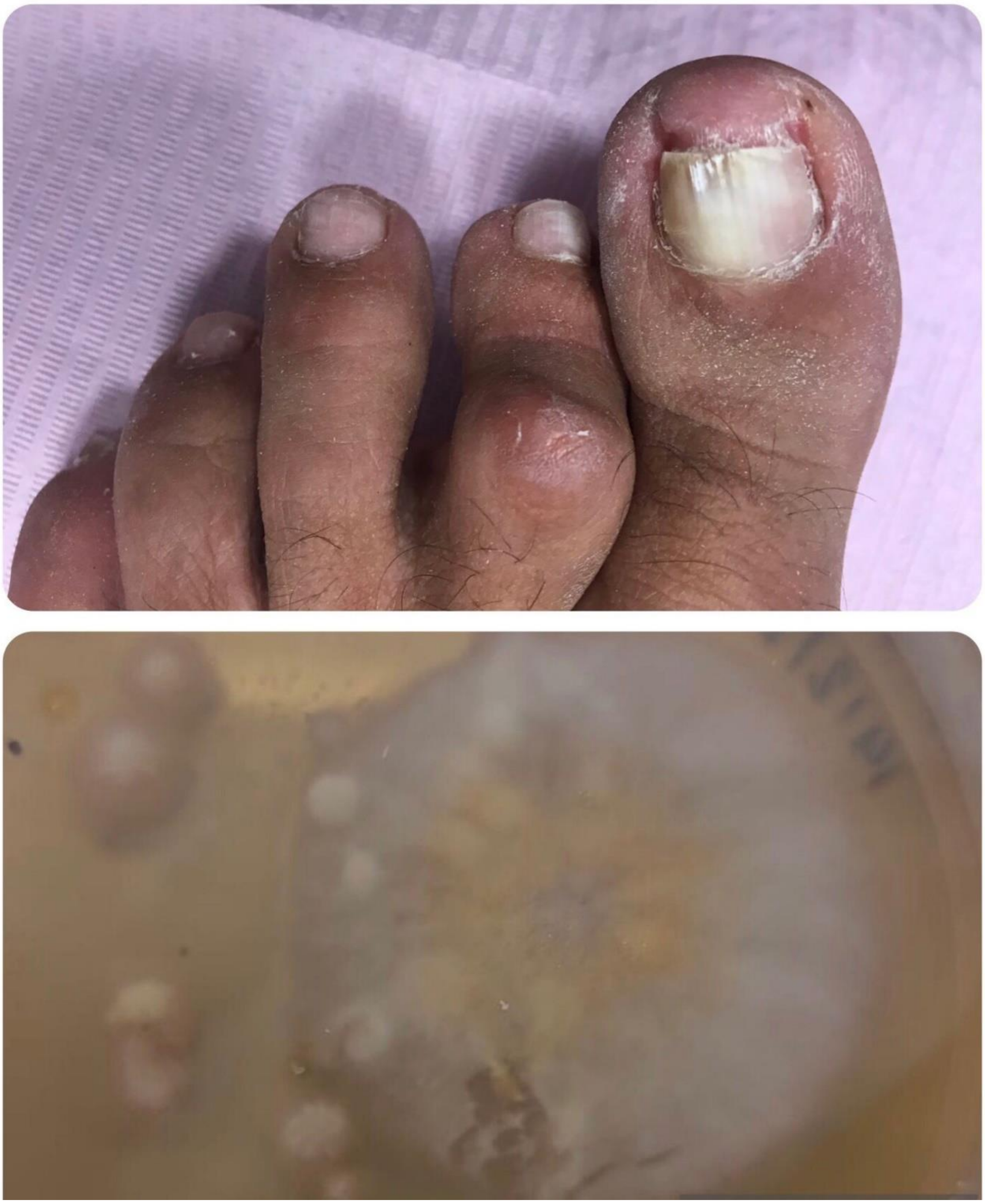

Figure S1: Onychomycosis compatible with Trichophytum Tonsurans according to the laboratory.

- **USUARIO 2**

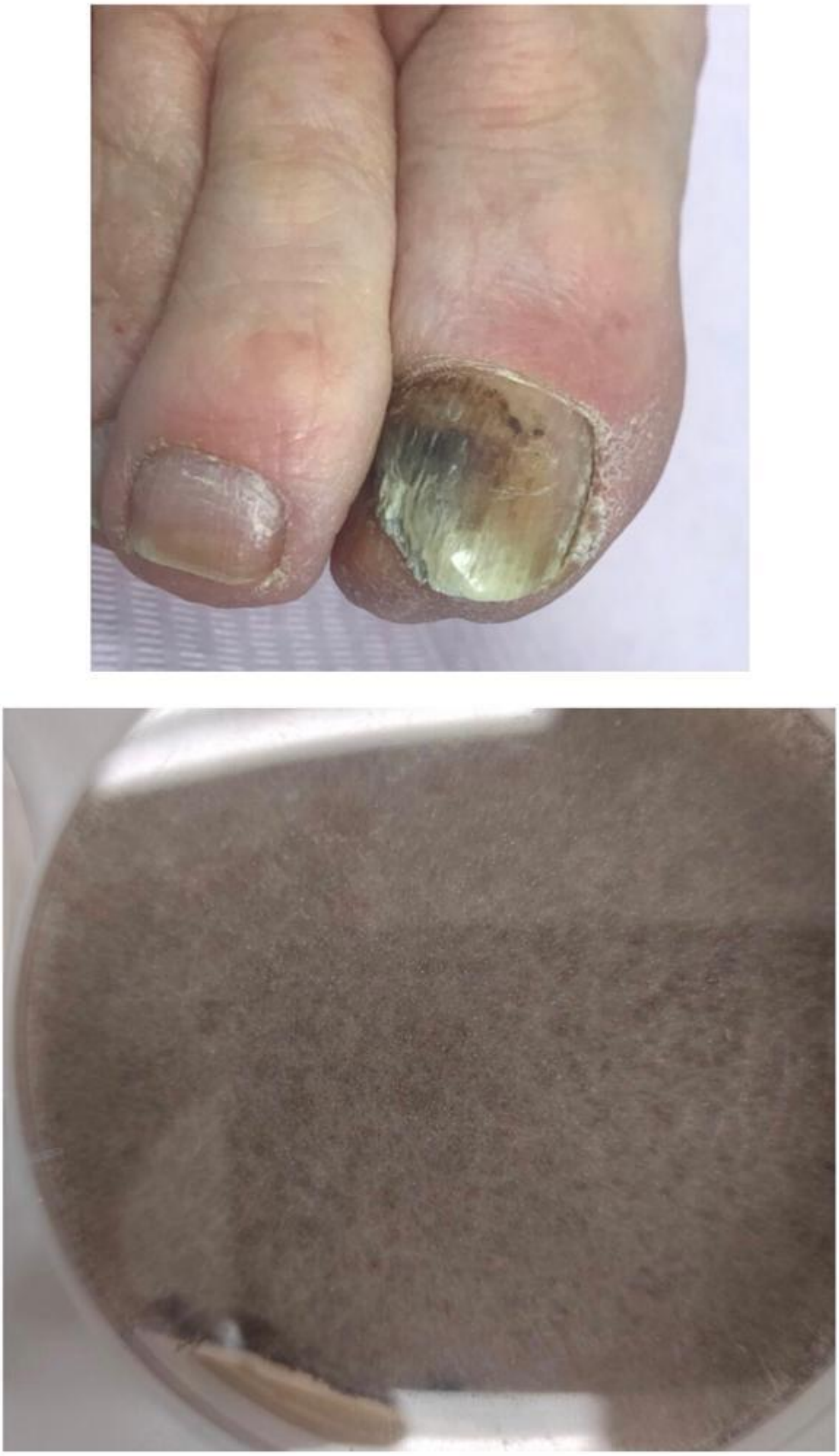

Figure S2: Onychomycosis compatible with Aspergillus Niger according to the laboratory.
